# The Role of Insulin Sensitivity in Lean Mass Changes During Weight Loss With or Without Exercise

**DOI:** 10.1002/oby.70010

**Published:** 2025-09-02

**Authors:** Ciera L. Bartholomew, Catia Martins, Barbara Gower

**Affiliations:** ^1^ Department of Nutrition Sciences University of Alabama at Birmingham (UAB) Birmingham Alabama USA

**Keywords:** aerobic exercise, insulin sensitivity, lean body mass, resistance exercise, skeletal muscle, weight loss

## Abstract

**Objective:**

This study aimed to investigate the association between baseline insulin sensitivity (S_I_) and changes in total lean mass (LM) and appendicular LM (ALM) during a diet‐induced weight loss program with or without exercise.

**Methods:**

We conducted a secondary data analysis from a controlled weight loss study in premenopausal women with overweight aged 21–41. Women were randomized into three groups: diet‐only, diet plus aerobic exercise, or diet plus resistance exercise. Body composition was assessed using dual‐absorptiometry X‐ray and S_I_ using an intravenous glucose tolerance test. Multiple linear regression was used to determine if baseline S_I_ was predictive of changes in total LM and ALM.

**Results:**

There were significant group‐S_I_ interactions on changes in total LM (*β* = 0.474, *p* = 0.003, adjusted *r* ^2^ = 0.371) and ALM (*β* = 0 0.462, *p* = 0.009, adjusted *r* ^2^ = 0.231) after adjusting for covariates, indicating that greater baseline S_I_ was associated with less LM loss only in women who did not exercise.

**Conclusions:**

Higher baseline S_I_ is associated with greater retention of LM when weight loss is induced with diet alone, but not when exercise (aerobic or resistance) is included. This reinforces the importance of including exercise in all weight loss interventions.

**Trial Registration:** ClinicalTrials.gov identifier: NCT00067873

**Study Importance**

- What is already known?
  ○ In individuals with obesity, ~20%–30% of weight loss comes from lean tissue, which can negatively affect energy expenditure and long‐term weight loss maintenance. Clinicians and researchers increasingly recognize the importance of muscle mass, and the need to preserve it during weight loss has grown with the use of weight loss medications.
  ○ Insulin is an anabolic hormone important for the regulation of muscle turnover. We have previously shown that greater sensitivity to insulin at baseline is associated with less loss of lean mass during a diet‐induced weight loss intervention. However, there is limited information on how insulin sensitivity and lean mass loss are related when exercise is part of the weight loss intervention.
- What does this study add?
  ○ This is the first study to evaluate the relationship between baseline insulin sensitivity, using an intravenous glucose tolerance test, and changes in lean mass during weight loss, in the presence or absence of a controlled exercise intervention including either aerobic or resistance training.
  ○ Higher baseline insulin sensitivity is associated with greater retention of lean mass when weight loss is induced with diet alone, but not when either aerobic or resistance exercise is included.
- How might these results change the direction of research or the focus on clinical practice?
  ○ Identifying individuals with lower insulin sensitivity prior to weight loss could enable tailored strategies to reduce lean mass loss.
  ○ Baseline insulin sensitivity may only impact changes in lean mass in the absence of exercise, reinforcing the importance of including exercise in all interventions designed to induce weight loss.

## 1 Introduction

Obesity prevalence worldwide has seen a twofold increase since 1990 [1]. This is a major public health concern given that obesity is associated with many of the leading causes of death, such as cancer and cardiovascular disease, as well as being the main risk factor for type 2 diabetes [2, 3]. Given these issues, treatment for obesity has become paramount. Weight loss is the primary method of obesity treatment and can be induced in one of three ways: behavior modification (diet and exercise), bariatric surgery, pharmacological treatment, or a combination of these approaches [4]. Each of these methods varies in terms of effectiveness, but one commonality is that they all result in a loss of fat free mass (FFM) [5, 6, 7, 8]. Ideally, weight loss should be derived almost exclusively from fat mass (FM); however, it has been estimated that in older adults with obesity, between 20% and 30% of the weight lost is comprised of FFM [9].

FFM is a heterogeneous body compartment that consists of all nonfat tissues, including skeletal muscle mass (SMM), bone mineral content (BMC), and digestive organs. The amount of FFM without BMC is often described as lean mass (LM). LM is often expressed as total body LM, which includes the torso and organs, and appendicular LM (ALM), which is the LM of the arms and legs, which reflects mostly SMM. Some have estimated that approximately 50% of FFM is SMM [10] and ALM has been estimated to account for ~75% of total body SMM [10]. LM loss is a common side effect of weight loss; however, its magnitude is dependent on multiple factors, namely age (greater loss with greater age), sex (greater loss in females than males), baseline BMI (greater loss with higher BMI), genetics, the degree of energy restriction (greater loss with greater restriction), exercise (the type and amount; greater loss with aerobic vs. resistance), protein content of the diet, and rate of weight loss [11, 12, 13, 14, 15, 16, 17, 18]. Minimizing LM loss is a concern given that LM has a U‐shaped association with all‐cause mortality [19], is associated with better quality of life in older adults [20], and plays a crucial role in determining energy expenditure and physical function [16, 21, 22]. As such, it is essential to understand how individuals can maintain LM or minimize its reduction during weight loss.

Insulin sensitivity is one factor that may influence changes in LM during weight loss. Insulin's primary role is to stimulate glucose uptake, and skeletal muscle is a major site for this action [23]. Insulin also acts as a potent anabolic stimulus for skeletal muscle by regulating amino acid availability and suppressing muscle protein breakdown [24, 25]. However, not all individuals respond to insulin the same way and might have different sensitivities to it. Insulin sensitivity is defined as the effect of insulin in stimulating glucose uptake by a tissue and is a marker of metabolic health [26].

Our group has previously shown that a greater baseline insulin sensitivity, assessed with an intravenous glucose tolerance test (IVGTT), was associated with less loss of LM during a controlled diet intervention [27]. Similarly, two other studies have shown associations between insulin resistance and greater loss of LM during weight loss [28, 29]. Taken together, these results indicate that there is a connection between measures of insulin sensitivity and changes in LM during weight loss. However, no study has looked at this relationship in the context of exercise. This is necessary as exercise is known to directly impact LM, and various forms of exercise impact LM preservation in distinct ways [9]. For instance, several studies have found that when resistance exercise is combined with caloric restriction, muscle mass can be preserved, while this is not always clearly demonstrated with aerobic exercise [9].

Considering that aerobic training and resistance training affect LM differently [9, 30], it is of relevance to explore how baseline insulin sensitivity contributes to LM loss during weight loss when aerobic or resistance exercise is added to an energy restricted diet. As such, the purpose of this analysis was to assess the association between baseline insulin sensitivity and changes in total LM and ALM during a weight loss study that included arms with and without supervised exercise (aerobic or resistance). We hypothesized that the impact of baseline insulin sensitivity on changes in LM would be altered by exercise.

## 2 Methods

### 2.1 Study Design

This was a weight loss intervention where participants were randomized to either diet‐only, diet plus aerobic exercise, or diet plus resistance training. The duration of the intervention was the time needed to reach a BMI of 25 kg/m^2^ or less.

### 2.2 Participants

Females ages 21–48 years, both White and African American, were recruited for this study. Inclusion requirements included: BMI between 27 and 30 kg/m^2^, being a nonsmoker, normal glucose tolerance (determined by a 2‐h serum glucose of < 140 mg/dL following 75 g oral glucose load), sedentary lifestyle (self‐report of exercising less than one time per week over the previous year), family history of obesity, and regular menstrual cycles. None of the women was taking oral contraceptives or any medications that could affect body composition or metabolism. All participants signed an informed consent form prior to testing. Further study details have been described elsewhere [30].

### 2.3 Protocol

All testing was conducted during the follicular phase of the menstrual cycle. Body composition, fat distribution, and metabolic outcomes were measured in a controlled setting during an inpatient stay at the General Clinical Research Center (GCRC) at baseline. Following discharge from baseline testing, the women commenced the weight loss phase, adhering to an 800 kcal/day diet designed to reduce body weight by > 10 kg, aiming to achieve an ideal weight target (BMI ≤ 25 kg/m^2^). Macronutrient distribution of the diet was the same for all three groups and consisted of 64% energy from carbohydrates, 21% from protein, and 15% from fat. Individuals randomized to the exercise arms began participating in supervised exercise. Participants were instructed to consume only the food provided by the GCRC, and body weight was monitored twice weekly until individuals achieved BMI ≤ 25 kg/m^2^.

### 2.4 Exercise Intervention

Exercise sessions were three times per week at a training facility dedicated to research on the University of Alabama at Birmingham's campus and lasted ~50 min. Training sessions were supervised by study staff.

#### 2.4.1 Aerobic Training

Aerobic training commenced with a warm‐up and stretching for 3–5 min. In the initial week of training, the participants engaged in a 20‐min session of sustained exercise at 67% maximum heart rate. After the first week, both the duration and intensity of the workouts were progressively elevated, reaching a point by the start of the 8th week where participants sustained continuous exercise at 80% of their maximum heart rate for 40 min. They maintained that level of intensity and duration until the end of the study. Exercise modalities included cycle ergometry, stair stepping, walking, and running.

#### 2.4.2 Resistance Training

Following a 5‐min warm‐up on either a treadmill or bike ergometer and a stretching routine lasting 3–5 min, participants performed exercises such as squats, leg extensions, elbow flexions, triceps extensions, lateral pull‐downs, bench presses, and lower back extensions. One set of 10 repetitions was performed during the first 4 weeks, after which two sets of 10 repetitions were performed for each exercise, with 2 min of rest between sets. The training regimen was structured to progress gradually, with intensity determined by lifting weights equivalent to 80% of an individual's one‐repetition maximum (1RM). Strength was evaluated in all exercises every 3 weeks, and adjustments in resistance were made based on the most current 80% of the 1RM an individual lifted. Methods used to assess strength have been described previously [30].

### 2.5 Outcome Measures

Measurements were conducted at baseline and post weight loss. All individuals were in the fasted state (at least 12‐h fast).

#### 2.5.1 Body Weight and Composition

Dual‐energy X‐ray absorptiometry (DXA) using a Lunar Prodigy densitometer (GE‐Lunar Corp., Madison, WI) was used to assess body composition. DXA scans were performed and analyzed with adult software version 1.33. Participants were instructed to dress in lightweight attire, remove any metal items, and lie flat on their backs with arms resting alongside their bodies during the total body scan. ALM was the sum of the LM at the arms and legs. Total body mass was the sum of FM, LM, and BMC. Change scores were calculated by subtracting the post‐weight loss measurement from baseline.

#### 2.5.2 IVGTT

An IVGTT was used to assess insulin sensitivity. At ≈07:00 after the subjects had fasted for 12 h, flexible intravenous catheters were inserted into the antecubital area of both arms. Participants received a 300 mg/kg solution with 50% dextrose, administered over 60 s, commencing at minute 0. Insulin (0.02 U per kilogram of body weight, Humulin; Eli Lilly, Indianapolis, IN) was infused between minutes 20 and 25. Blood was collected in the fasted state (−30, −20, −15, −5, and −1) and at times 2, 3, 4, 5, 6, 8, 10, 12, 15, 19, 20, 21, 22, 24, 26, 28, 30, 35, 40, 45, 50, 55, 60, 70, 80, 100, 120, 140, 180, 210, and 240 min post glucose administration. The acute insulin response to glucose (AIRg) was the incremental area under the curve of insulin from 0 to 10 min after glucose administration. AIRg was calculated using the trapezoidal method.

#### 2.5.3 Hormone Analysis

All analyses were conducted in the Core Laboratory of University of Alabama at Birmingham's GCRC and Clinical Nutrition Research Center. Glucose was measured using an Ektachem DT II System (Johnson and Johnson Clinical Diagnostics, Rochester, NY). In the Core Laboratory, this analysis has a mean intra‐assay CV of 0.61% and a mean inter‐assay CV of 2.56%. Insulin was assayed in duplicate 100 μL aliquots using double‐antibody radioimmunoassay (Linco Research Inc., St. Charles, MO). In the Core Laboratory, this assay has a sensitivity of 3.35 μIU/mL, a mean intra‐assay CV of 3.49%, and a mean inter‐assay CV of 5.57%.

#### 2.5.4 Mathematical Modeling

An insulin sensitivity index (S_I_) was derived from glucose and insulin values from the IVGTT. Glucose and insulin values were entered into the MINMOD computer program (version 3, Richard N. Bergman) for determination of S_I_ [31].

### 2.6 Statistical Analysis

Statistical analyses were performed using SPSS version 22 (SPSS Inc., Chicago, IL). Participants were included in the analyses if they had data for the outcome variables of interest both at baseline and post weight loss, as well as data for each covariate. The statistical significance was set at *p* < 0.05, and data were presented as means and standard deviations (SD). Each variable was assessed for normality using histograms and quantile‐quantile (Q–Q) plots. Any variables not normally distributed were log transformed prior to analyses. Paired *t* tests were used to assess mean changes over time in anthropometric measures from baseline, and ANCOVA was used to determine group differences in total LM and ALM post intervention after adjusting for baseline values. Significant main effects were further explored using Dunnett's T3 post hoc tests to identify specific group differences. Of the 226 individuals originally enrolled in the study, 127 had data on the variables of interest and were included in the analyses. Participants were removed due to missing data or being considered outliers. Outliers were defined as observations with residuals greater than 3 SD from the mean of the residuals.

**FIGURE 1:**
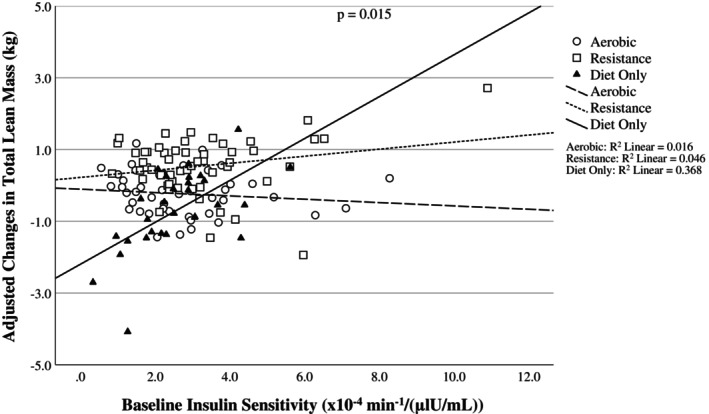
Scatterplot for the association between baseline insulin sensitivity (S_I_) and changes in total lean mass (LM) during weight loss after adjusting for covariates. Changes in total LM were calculated by subtracting post‐weight loss from baseline values.

**FIGURE 2:**
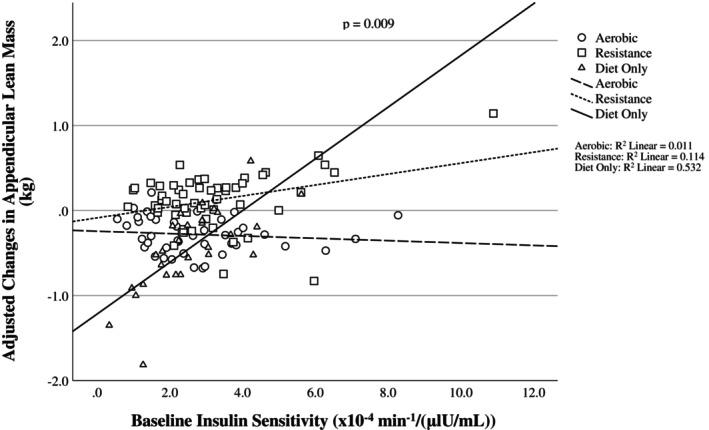
Scatterplot for the association between baseline insulin sensitivity (S_I_) and changes in appendicular lean mass (ALM) during weight loss after adjusting for covariates. Changes in ALM were calculated by subtracting post‐weight loss from baseline values.

The associations between baseline S_I_ and changes in total LM and ALM after weight loss were determined using multiple linear regression. Standardized *β* coefficients and 95% confidence intervals (CI) are reported. Model assumptions were assessed by inspecting the residuals and skewness statistics for normality and plots of residuals by predicted values for homoscedasticity. Multicollinearity was tested using the variance inflation factors for each variable in the model. Weight loss, baseline FM, baseline AIRg, baseline LM, race, intervention group, and S_I_*group interaction were used as covariates. Adjusted values were calculated using a multiple linear regression model with all the covariates included and are displayed in Figures 1 and 2. Baseline FM was included given that it is inversely associated with S_I_ and FFM loss during weight loss [32]. Baseline AIRg was included in the models due to the well‐known inverse association between S_I_ and AIRg and due to the potential effect of insulin itself on the changes in LM observed during weight loss.

## 3 Results

A total of 127 participants were included in the analyses, with 29 in the diet‐only group, 55 in the resistance exercise group, and 43 in the aerobic exercise group (Table 1). Baseline BMI was ~28 kg/m^2^ in all three groups. Average age was 34.5 ± 6.2 years, and 51% of the sample was African American in the two exercise groups and 41% in the diet‐only group. Duration of the weight loss intervention was reported as days to achieve BMI < 25 kg/m^2^. This was 146 ± 44 days in the diet‐only group, 157 ± 76 days in the resistance group, and 160 ± 74 days in the aerobic group.

**TABLE 1:** General characteristics and anthropometrics over time by group.

Variables	Diet only group (*n* = 29)			Resistance exercise group (*n* = 55)			Aerobic exercise group (*n* = 43)		
	Baseline	Post‐weight loss	*p*	Baseline	Post‐weight loss	*p*	Baseline	Post‐weight loss	*p*
Age (years)	35.5 ± 5.6			33.4 ± 5.8			35.3 ± 6.9		
Race % (AA)	41.4%			51.1%			51.2%		
S_I_ (× 10^−4^ min^−1^/(μlU/mL))	2.5 ± 1.1			3.1 ± 1.7			2.7 ± 1.6		
AIRg (μlU/mL × 10 min)	617 ± 384			750 ± 430			788 ± 552		
Days to BMI < 25 kg/m^2^	146 ± 44			157 ± 76			160 ± 74		
BMI (kg/m^2^)	28.2 ± 1.2	23.8 ± 1.1	< 0.00 1	28.1 ± 1.1	23.9 ± 1		28.3 ± 1.4	23.8 ± 1.1	< 0.001
Body weight (kg)	77 ± 7.5	65.5 ± 6.4	< 0.001	77.5 ± 7.7	66.3 ± 6.6	< 0.001	76 ± 7	64 ± 6.3	< 0.001
Fat mass (kg)	33.9 ± 5.1	22.2 ± 4.6	< 0.001	33.5 ± 5.2	22 ± 4.3	< 0.001	33.5 ± 5	22.7 ± 4.6	< 0.001
LM (kg)^a^	41.2 ± 3.8	40.5 ± 3.6	0.049	41.1 ± 4	42.2 ± 4	< 0.003	40.4 ± 4.9	39.4 ± 3.5	0.173
Appendicular LM (kg)^a^	18.8 ± 2.3	18.3 ± 2.3	0.022	19.3 ± 2.1	19.4 ± 2.2	0.256	18.4 ± 1.9	18.2 ± 2	0.010
LM loss (kg)		−0.77 ± 2.2			0.64 ± 1.4			−0.35 ± 1.4	
ALM loss (kg)		−0.43 ± 0.97			0.12 ± 0.72			−0.30 ± 0.76	
FFM loss (kg)		−0.83 ± 2.6			0.52 ± 1.4			−0.47 ± 1.33	

*Note*: Data presented as mean ± SD and percentages. *p* < 0.05 indicate a statistically significant change over time within each group. Differences were seen between the resistance exercise group and the aerobic exercise and diet only groups. There were no differences between aerobic and diet only groups.

Abbreviations: AA, African American; AIRg, acute insulin response to glucose; ALM, appendicular lean mass; FFM, fat free mass; LM, lean mass; S_I_, insulin sensitivity.

^a^ Indicates that there were statistically significant group differences in post‐weight loss LM and ALM after adjusting for baseline measures as seen by the post hoc analysis.

Individuals lost on average 11.4 ± 2.4 kg of body weight. A significant increase in total LM was seen in the resistance exercise group (*p* < 0.001), while no changes were seen in the other groups. ALM decreased from baseline in the diet‐only and aerobic exercise groups (*p* = 0.022 and *p* = 0 0.010, respectively), but not in the resistance exercise group. Significant differences in total and ALM post weight loss were seen among groups, after adjusting for baseline values (*f* = 9.32, *p* < 0.001; *f* = 6.42, *p* = 0.002). Post hoc tests revealed that total LM was significantly higher in the resistance group compared with aerobic (*M*_diff_ = 0.87, 95% CI: [0.186, 1.57], *p* = 0.008) and diet‐only groups (*M*_diff_ = 1.31, 95% CI: [0.320, 2.24], *p* = 0.006). Similarly, ALM was significantly higher in the resistance compared with aerobic (*M*_diff_ = 0.380, 95% CI: [0.044, 0.716], *p* = 0.021) and diet‐only groups (*M*_diff_ = 0.546, 95% CI: [0.043, 1.05], *p* = 0.030). The aerobic and diet‐only groups were not significantly different from one another in terms of total LM or ALM post intervention.

Multiple linear regression analyses for total LM and ALM are shown in Tables 2 and 3. As seen in Table 2, there was a significant group*S_I_ interaction on changes in total LM (*β* = 0.410, *p* = 0.015) after adjusting for race, group, total weight loss, and baseline levels of FM, LM, AIRg, and S_I_ (adjusted *r* ^2^ = 0.322). Similarly, as shown in Table 3, there was a significant group*S_I_ interaction on changes in ALM (*β* = 0 0.468, *p* = 0.009) after adjusting for race, group, total weight loss, and baseline levels of ALM, AIRg, and S_I_ (adjusted *r* ^2^ = 0.231). The interaction suggests that the association of S_I_ with LM and ALM differed with group, being positive in the diet‐only and diet plus resistance exercise groups but negative in the diet plus aerobic exercise group. These results can be seen visually in Figures 1 and 2.

**TABLE 2:** Multiple linear regression model predicting changes in total lean mass.

Predictor	*β* coefficient (95% CI)	*p*	Adjusted *R* ^2^
Model		< 0.001	0.322
Race	0.111 (−0.193 to 0.897)	0.203	
Group	0.363 (0.580 to 2.15)	< 0.001	
Weight loss (kg)	0.445 (0.171 to 0.406)	< 0.001	
Baseline FM (kg)	0.107 (−0.023 to 0.089)	0.239	
Baseline LM (kg)	−0.136 (−0.118 to 0.007)	0.080	
Baseline AIRg (μlU/mL × 10 min)	0.081 (0.000 to 0.001)	0.383	
Baseline S_I_ (× 10^−4^ min^−1^/(μlU/mL))	−0.170 (−0.489 to 0.150)	0.295	
Group*S_I_ interaction	0.410 (0.036 to 0.330)	0.015	

*Note*: Variance inflation factors < 5. Race: 0 = EA (European American), 1 = AA (African American); Group: 1 = Aerobic, 2 = Resistance, 3 = Diet only.

Abbreviations: AIRg, acute insulin response to glucose; FM, fat mass; LM, lean mass; S_I_, insulin sensitivity.

**TABLE 3:** Multiple linear regression model predicting changes in appendicular lean mass.

Predictor	β coefficient (95% CI)	*p*	Adjusted *R* ^2^
Model		< 0.001	0.231
Race	0.155 (−0.059 to 0.546)	0.114	
Group	0.377 (0.290 to 1.12)	0.001	
Weight loss (kg)	0.306 (0.045 to 0.151)	< 0.001	
Baseline ALM (kg)	−0.077 (−0.090 to 0.033)	0.367	
Baseline AIRg (μlU/mL × 10 min)	0.002 (0.000 to 0.000)	0.983	
Baseline S_I_ (× 10^−4^ min^−1^/(μlU/mL))	−0.199 (−0.266 to 0.070)	0.249	
Group*S_I_ interaction	0.468 (0.027 to 0.181)	0.009	

*Note*: Variance inflation factors < 5. Race: 0 = EA, 1 = AA; Group: 1 = Aerobic, 2 = Resistance, 3 = Diet only.

Abbreviations: AIRg, acute insulin response to glucose; ALM, appendicular lean mass; S_I_, insulin sensitivity.

## 4 Discussion

The present analyses were designed to determine the association between baseline S_I_ and changes in LM during a weight loss intervention that included arms with diet‐only and diet plus aerobic or resistance exercise. On average, individuals lost 11.4 ± 2.4 kg of body weight. The diet‐only and the aerobic exercise groups experienced significant reductions in ALM, while the resistance group experienced a significant increase in total LM. There was a significant group*S_I_ interaction for changes in total LM and ALM such that an elevated baseline S_I_ was associated with greater LM retention, but only in the diet‐only group. These results suggest that the influence of S_I_ on LM retention during weight loss is not as strong when exercise is included. Visual inspection of the figures suggests that the differences between groups are manifested at the lower and upper ends of S_I_.

These results are consistent with previous longitudinal studies. Our research group has shown that baseline S_I_ was a predictor of changes in total LM and ALM during a controlled weight loss intervention [27]. Additionally, Wong and colleagues examined data from two controlled weight loss studies and described that higher HOMA‐IR levels (lower insulin S_I_) were associated with greater LM loss [28]. In addition, previous cross‐sectional studies have shown that low LM is associated with greater insulin resistance [33, 34, 35]. However, none of these studies included exercise, and only one of the longitudinal interventions specifically assessed baseline S_I_ using a valid and reliable method. The study being presented here not only assessed baseline S_I_ and body composition using advanced techniques, but it also included two exercise modalities, individually prescribed and monitored.

Energy balance is essential in determining the fate of ingested amino acids, as it allows the amino acids to be used primarily for protein synthesis, rather than being diverted to meet energy demands [36]. In the context of weight loss and caloric restriction, protein is degraded and whole‐body protein breakdown and synthesis are reduced, especially if the duration of caloric restriction is prolonged [37]. However, when exercise is added to caloric restriction, the balance between protein use for energy versus non‐energy functions can be altered. Aerobic training and resistance training have been shown to impact LM differently during weight loss [9, 30]. When compared to resistance exercise, aerobic exercise can have a catabolic effect on SM. Resistance training, on the other hand, is an important countermeasure to LM loss and often results in either maintenance or lower loss of LM during weight loss [30].

In this study, the association between baseline S_I_ and changes in LM was the strongest in the diet‐only group. The weaker association in the resistance exercise group can potentially be explained by the fact that this group experienced an increase in total LM and no changes in ALM over time, unlike the other groups. Resistance exercise is more anabolic than aerobic exercise [38], with protein synthesis increasing more than protein breakdown in response to this type of exercise [39]. On the other hand, several studies have shown that whole body protein breakdown, as determined by leucine disappearance, is increased during aerobic exercise [39]. It is possible that the independent influence of aerobic and resistance exercise on protein metabolism masked the relationship between baseline S_I_ and changes in total LM and ALM seen in the diet‐only group.

The present analyses showed that a higher baseline S_I_ has a protective effect on LM when weight loss is achieved with diet‐only. This could be because insulin influences amino acid flux in and out of the muscle. During procedures involving insulin infusion, plasma amino acid levels fall [39], and muscle protein breakdown is suppressed [40], suggesting that insulin modulates muscle protein turnover [40]. The lack of association between baseline S_I_ and LM loss in the exercise groups suggests that exercise overrides, or masks, the influence of S_I_ on muscle protein metabolism.

In this study, both total LM and ALM increased in the resistance group, albeit not significantly in the case of ALM. In contrast, ALM decreased in the aerobic and diet‐only groups. Given that most of the exercises were done using the arms and legs, it is surprising that the increase in ALM was not significant. One explanation could be the limitations of DXA in measuring lean tissue. There is a large water component in the lean soft tissue assessed by DXA [41], and alterations in hydration could impact measures [42]. Exercise also alters muscle glycogen content, which impacts total body water and subsequently LBM assessed by DXA [43]. Future studies are needed to better characterize the nature of LM changes during weight loss and how they are affected by S_I_ and exercise modality.

The proportion of weight lost as LM in the present study was smaller than what has been previously described in diet‐induced weight loss interventions [9, 44]. The fact that our secondary analysis was performed in all groups combined (diet only, diet plus aerobic training, and diet plus resistance training) might explain some of the discrepancies, as exercise, in particular resistance training, preserves LM, as previously shown in this cohort [30]. However, the smaller proportional loss of LM was also seen in the diet only group. The high protein content of the diet (21% of the total energy), the low age of our participants, and the inclusion of women only (both African American and European American) might have additionally contributed to the differences in proportional LM loss across weight loss interventions.

This study has both strengths and limitations. One strength is that all exercise training was supervised by trained personnel and all the food was provided, with protein intake being similar across groups. Additionally, validated and reliable methods were used to assess S_I_ and body composition. A limitation of this study is that no causal relationship between baseline S_I_ and LM loss can be established. Furthermore, the present findings need to be replicated in other populations, namely older adults, men, and those taking new obesity medications. Future studies should assess SMM using methods like magnetic resonance imaging. More research is also needed to better understand the mechanisms through which S_I_ regulates changes in LM during weight loss.

In conclusion, the findings of this study reinforce the importance of adding exercise to weight loss interventions involving energy restriction. In those unable to exercise, evaluating their baseline S_I_ may allow for personalized nutrition to minimize LM loss.

## Author Contributions

C.L.B., C.M., and B.G. produced the research question for this secondary data analysis and were involved in the writing of the manuscript.

## Conflicts of Interest

The authors declare no conflicts of interest.

## Data Availability

The data that support the findings of this study are available from the corresponding author upon reasonable request.
